# Biochemical and Functional Studies of Lymphoid-Specific Tyrosine Phosphatase (Lyp) Variants S201F and R266W

**DOI:** 10.1371/journal.pone.0043631

**Published:** 2012-08-27

**Authors:** Jing Liu, Ming Chen, Rong Li, Fan Yang, Xuanren Shi, Lichao Zhu, Hong-Mei Wang, Wei Yao, Qiji Liu, Fan-Guo Meng, Jin-Peng Sun, Qi Pang, Xiao Yu

**Affiliations:** 1 Key Laboratory for Experimental Teratology of the Ministry of Education, Shandong University School of Medicine, Jinan, Shandong, China; 2 The 309^th^ Hospital of PLA, Beijing, China; 3 Yangtze Delta Region Institute of Tsinghua University, Zhejiang, China; 4 Shandong Provincial Hospital, Shandong University, Jinan, Shandong, China; University of Leuven (KU Leuven), Faculty of Medicine, Belgium

## Abstract

The Lymphoid specific tyrosine phosphatase (Lyp) has elicited tremendous research interest due to the high risk of its missense mutation R620W in a wide spectrum of autoimmune diseases. While initially characterized as a gain-of-function mutant, R620W was thought to lead to autoimmune diseases through loss-of-function in T cell signaling by a recent study. Here we investigate the biochemical characters and T cell signaling functions of two uncharacterized Lyp variants S201F and R266W, together with a previously characterized Lyp variant R263Q, which had reduced risk in several autoimmune diseases, including systemic lupus erythematosus (SLE), ulcerative colitis (UC) and rheumatoid arthritis (RA). Our kinetic and functional studies of R263Q polymorphism basically reproduced previous findings that it was a loss-of-function mutant. The other variant S201F reduced Lyp phosphatase activity moderately and decreased Lyp function in T cell slightly, while R266W severely impaired phosphatase activity and was a loss-of-function variant in T cell signaling. A combined kinetic and structure analysis suggests that the R266W variant may decrease its phosphatase activity through perturbing either the Q-loop or the WPD loop of Lyp. As both R266W and R263Q significantly change their phosphatase activity and T cell functions, future work could be considered to evaluate these mutants in a broader spectrum of autoimmune diseases.

## Introduction

Protein tyrosine phosphorylations regulated by protein tyrosine kinases (PTKs) and protein tyrosine phosphatases (PTPs) are essential signal transduction events mediating the immune response [1], [2]. Disregulation of either PTKs or PTPs leads to the abnormal immune response and correlates to human disease development [3], [4]. Most importantly, PTPN22, which encodes lymphoid-specific tyrosine phosphatase (Lyp), attracts tremendous attentions due to the linkage of its R620W single nucleotide polymorphism (SNP) to many autoimmune diseases, including Type 1 Diabetes [5], [6], rheumatoid arthritis [7], [8], and systemic lupus erythematosus [9], [10]. Therefore, intensive efforts have been input to investigate Lyp’s cellular function and its underlying mechanism in autoimmune diseases [11], [12], [13], [14], [15], [16].

Human Lyp was first identified in 1999, with 90% homology to murine phosphatase PEP in its phosphatase catalytic domain [17]. Lyp and its murine homologue PEP are negative regulators in T cell signaling through direct dephosphorylation of Lck and ZAP70 kinases [17], [18], [19], [20]. Lyp also associates with CSK, an Lck negative regulator, through the interaction of its first C-terminal poly-proline (P1) region with SH3 domain of CSK [21], [22], [23]. The disease related mutation R620W, which resides in the P1 region, disrupts this interaction and is firstly reported as a gain-of-function mutation in regulating T cell function [5]. It was observed that less interleukin-2 was secreted from T cells with R620W allele. These studies indicate selectively inhibiting Lyp activity may be considered to develop new treatment for autoimmune diseases [5], [12], [13]. A specific salicylic acid-based inhibitor was identified through our previous biochemical studies, and it could rescue impaired B cell signaling in Lyp620W- expressing B cell [12], [24]. Conversely, recent research argues that R620W decreased Lyp expression level and causes disease through an impaired T cell function, raised the question whether Lyp can be a therapeutic target [16].

Besides Lyp R620W mutation, human genomics studies have identified several missense polymorphisms which did not display significant correlation to cause immune diseases [25], [26]. One of the variants, R263Q, which was identified as a loss-of-function mutation, was found to reduce the risk of several autoimmune diseases, including systemic lupus erythematosus, ulcerative colitis and rheumatoid arthritis, but increase susceptibility to infectious disease like pulmonary tuberculosis (PT) [25], [27], [28], [29]. These results suggest the important role of Lyp polymorphism in different autoimmune diseases. Besides R620W and R263Q variants, genetics and clinical studies have accumulated more Lyp polymorphisms with few investigations on their autoimmune disease relationship. A deeper insight of Lyp polymorphism effects on its activity and function will improve our understanding of its potential relationship to autoimmune diseases.

In our previous study, we purified the catalytic domain of Lyp and solved its crystal structure together with either a specific inhibitor or peptide substrates [12], [14]. The crystal structure revealed that Lyp catalytic domain assumed a classic tyrosine phosphatase folding with a specific insert at N-terminal, which was a determinant of Lyp substrate specificity. In this work, we biochemically characterized two new Lyp polymorphisms, S201F and R266W, together with a previously characterized variant R263Q. Basically, we reproduced the previous biochemical and cellular phenotype of the R263Q polymorphism [25]. In addition, we found that R266W significantly decreased its phosphatase activity toward several substrates, including the small artificial substrate pNPP, the Lck phosphor-peptide 394, and the phosphor-Src protein catalytic domain. Another Lyp variant, S201F did decrease the activity toward pNPP, but only slightly impaired its activity towards the Lck phosphor-peptide and the purified phosphor-Src protein. In consistent with these biochemical results, R266W impaired Lyp function significantly in negatively regulating T cell signaling in cells while S201F displayed a moderate decreased effect. Future work could be considered to evaluating the association of these Lyp polymorphisms with a broad spectrum of autoimmune diseases, and see how they relate to our biochemical and T cell functional data.

## Results

**Figure 1:**
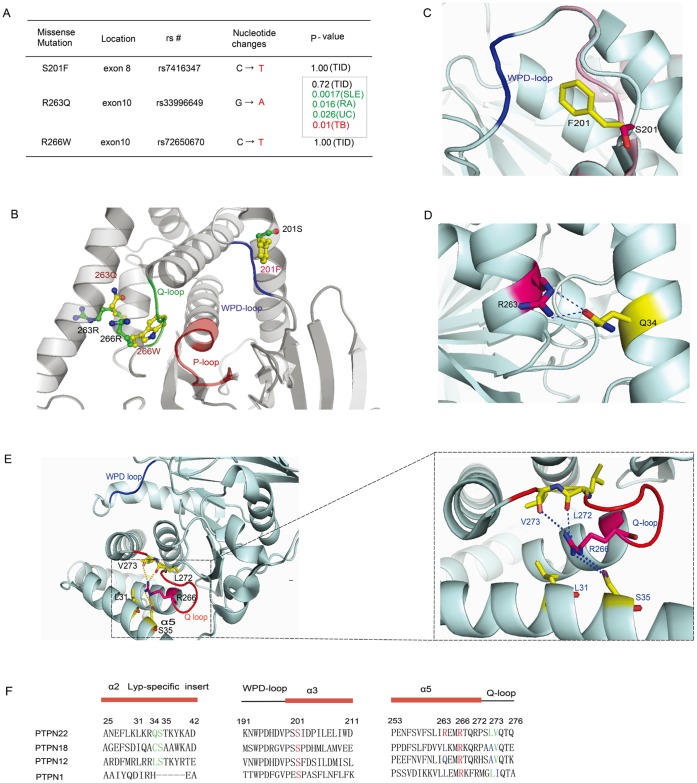
Disease association, structure representation, and sequence alignment of 3 Lyp variants S201F, R263Q and R266W. (A) Association analysis of S201F, R263Q and R266W in T1D patients and association of R263Q with RA, TB, UC and SLE patients. (B) Structural representation of Lyp variants S201F, R263Q and R266W. The figure was prepared with Pymol using the coordinates from PDB code 3OLR. P-loop, WPD-loop and Q-loop are depicted in red, blue and green, respectively. (C) Structure model of S201F. The bulky Phe instead of hydrophilic Ser at position 201 may affect WPD loop movement. The figure was prepared with Pymol using the coordinates for PDB code 3OLR and 2QCJ. (D) Structure representation of R263. R263 faces to Lyp specific insert, and the guanine group of R263 interacts with the side chain of Q34 through hydrogen bonds. The figure was prepared with Pymol using the coordinates for PDB code 3OLR. (E) Structure model of R266. R266 resides in the α5 helix adjacent to Q loop. The side chain of R266 forms hydrogen bonds with the main-chain amides of L272 and V273, and the side chain of S35. R266 also makes hydrophobic interaction with L31 and V273. The figure was prepared with Pymol using the coordinates for PDB code 3OLR. (F) Sequence alignment of α2′-Lyp-specific insert, WPD-loop-α3, and α5-Q-loop with corresponding regions from PTPN12, PTPN18 and PTPN1. S201, R263 and R266 are highlighted in red. Interacting residues are represented in green. The unconservative residues are shown in blue.

We have looked up SNPs of PTPN22 on the collective NCBI’s Entrez SNP system (http://www.ncbi.nlm.nih.gov/snp). Among them, we investigated the phosphatase activity of S201F (C602T, rs74163647), R266W (C796T, rs72650670) together with R263Q (G788A, rs33996649), which ranked top three of population in the sequenced pools of DNA from T1D patients and controls [26]. Previous clinical studies indicated that these variants were not related to T1D, while R263Q reduced the risk of SLE, UC and RA [25], [28](Fig. 1A). We then examined these three variants from an evolutionary prospective. As reported by a recent study, the amino acid at Lyp 263 position in human being is a unique R, while all other mammalian species contain an invariable Q. Different from R263Q polymorphism, Ser at postion 201 and Arg at position 266 are highly conserved in vertebrates, in consistent with the finding that they are rare mutations in homo sapiens (Fig. S1). Then we checked the location of these mutations in the crystal structure of Lyp catalytic domain. Both Arg 263 and Arg 266 reside in helix alpha 5, just before the Q loop, and Ser 201 locates at the beginning of alpha3 helix, behind the WPD loop (Fig. 1B–1E). As WPD loop and Q loop provide conserved essential catalytic residues of D195 and Q274 which are required for efficient hydrolysis of PTP substrates (scheme1), we investigated whether these variants directly affect Lyp phosphatase activities.

We overexpressed the catalytic domain (1–294) wild type and three Lyp variants, 266W, 263Q and 201F in E-coli, and purified these proteins to near homogenity by passing the lysates through Ni^2+^-affinity column and CM cation exchange column. As shown in figure S2, these variants displayed good protein yield and purity as the wild type (Fig. S2).

**Figure 2:**
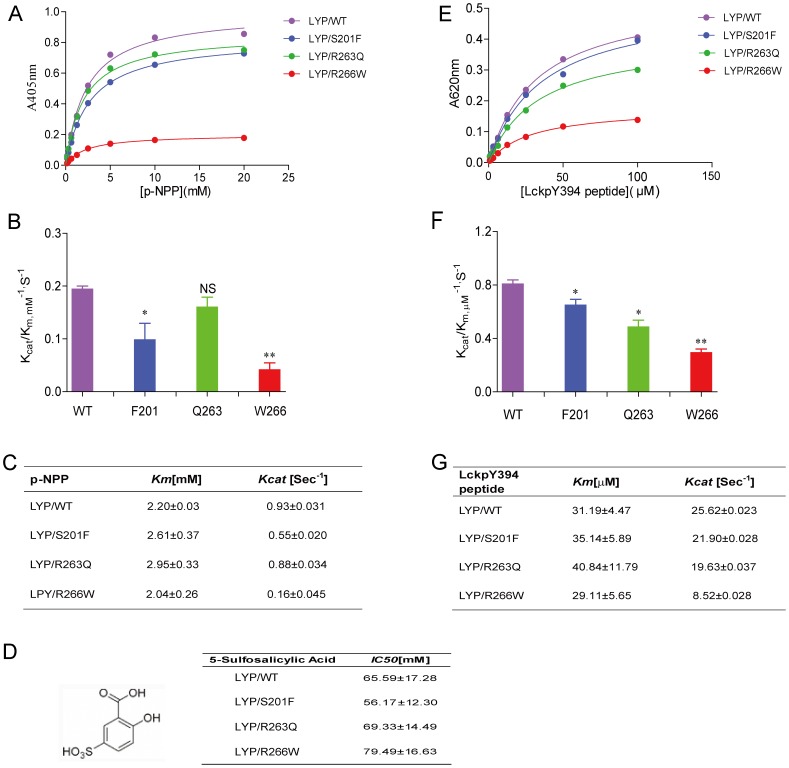
Kinetic analysis of the phosphatase activity of Lyp catalytic domain wild type and S201F, R263Q and R266W mutants toward pNPP and phosphor-peptide. (A) pNPP hydrolysis with Lyp catalytic domain domain and 3 mutants. Data were fitted to the Michaelis-Menten equation. (B–C) Kinetics parameters for the wild-type and the mutants of Lyp toward pNPP. All experiments were repeated four times. * represents P<0.05; ** represents P<0.01. (D) IC50 values of 5- sulfosalicylic acid toward Lyp wild type and variants. (E) Lyp catalyzed phosphate release of a 9 amino acid phosphor peptide derived from Lck 394 phosphorylation site (EDNEpYTARE). Data were fitted to the Michaelis-Menten equation. (F–G) Kinetics parameters for the wild-type and the mutants of Lyp toward EDNEpYTARE. Data showed the mean of three independent experiments. *P<0.05; ** P<0.01.

With the purified proteins, we checked whether these variants affected their intrinsic phophatase activity toward pNPP, a small aryl phosphate substrate, through steady state kinetic analysis. As a result, the Km of Q263 increased slightly compared with the wild type R263 while the Kcat didn’t change much. The Kcat/Km of Q263 toward pNPP, which reflected the intrinsic phosphatase activity, didn’t show statistics difference compared to the wild type R263. However, Kcat/Km values of the other two Lyp mutants, F201 and W266 decreased significantly to 1/2 and 1/5 compared to the wild type respectively (Fig. 2B). The changes of the catalytic activity of these variants are mainly due to Kcat, but not Km, which were shown in Fig. 2A and 2C. As Km may reflect the substrate binding to the enzyme while Kcat are determined by rate limiting steps such as K2 and K3 (shown in the scheme 1), these kinetic results suggest that these variants have little effects on changing the active site shape, but may influence the positioning of WPD and Q loops during catalysis, leading to a decreased K2 or K3 value. To further investigate whether these variants have effects on protein active site folding, we also determined the inhibition abilities of 5-sulfosalicylic acid towards Lyp variants. The competitive inhibitor 5-sulfosalicylic acid had a similar IC50 towards both wild type and 3 Lyp variants, proving that these variants maintained a similar active site as wild type (Fig. 2D).

Scheme 1:
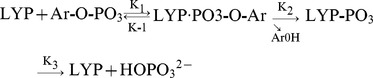



Lyp substrate specificity partially resides in its catalytic domain, which was revealed by our previous study using an inverse alanine library screening and crystallographic analysis. To determine whether Lyp polymorphisms affect Lyp substrate specificity towards primary peptide sequence, we examined these variants’ activities toward a 9-amino acid phosphor-peptide derived from the Y394 phosphorylation site of Lck, a physiological substrate of Lyp. Polymorphisms of F201, Q263 and W266 had reductions on Kcat/Km values about 20%, 40% and 60% respectively, all with statistics significance. Interestingly, F201 had a better activity towards the peptide substrate compared to pNPP, while Q263 had a decreased activity towards peptides, indicating F201 preferred its physiological substrate over the small artificial substrate while Q263 lost the ability for physiological substrate recognition.

**Figure 3:**
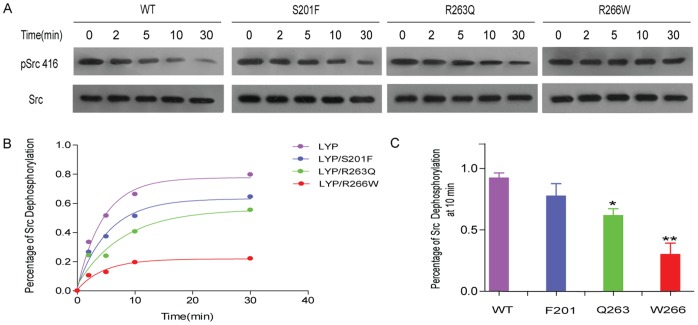
Dephosphorylation of pSrc416 by Lyp wild type and mutants. (A) Time dependence of pSrc416 dephosphorylation by Lyp wild type, S201F, R263Q and R266W. The phosphorylation level of the Src protein at 416 position was monitoted by Western blotting. (B) The dephosphorylation levels were quantified with Image J software, and plotted against the different time. (C) Statistical analysis of Lyp catalyzed pSrc416 dephosphorylation at 10 min. All experiments were repeated in triplicate. *P<0.05; ** P<0.01.

The observations that some Lyp variants like S201F displayed relatively different substrate selectivity towards Lck 394 phosphor-peptide and pNPP elicited our interests that these mutants might have additional effects on their physiological substrates. One explanation is that these polymorphisms might affect the protein surface responsible for substrate recognition other than the active sites. As a potent negative regulator of T cell signaling, Lyp could directly dephosphorylate the Lck at 394 position [12], [18]. Without enough quantities of recombinant phosphorylated Lck protein prepared in vitro, we used purified recombinant phoshporylated Src protein for kinetic analysis, exploiting its highly sequence identity and identical residues surrounding the activated tyrosine phosphorylation sites. We monitored the dephosphorylation of Src at 416 position by Lyp with western blotting. As shown in Fig. 3A, Lyp wild type efficiently dephosphorylated Src at 416 position, with almost half of substrate hydrolysation occoured within 5 min. The F201 and Q263 reached the half dephosphorylation at 10 min and 30 min respectively (Fig. 3A & 3B). The R266 significantly decreased its activity toward the purified phosphorylated Src protein, with only 20% dephosphorylation occured after 30 min. Once we compared the dephosphorylation at 10-minute-time point, Q263 and W266 displayed a significant reduction on their activity toward the phosphporylated Src protein while F201 didn’t show statistic significance (Fig. 3C). These kinetic results on the phosphor-Src protein were similar to the data that acquired from Lck394 phosphor-peptide.

**Figure 4:**
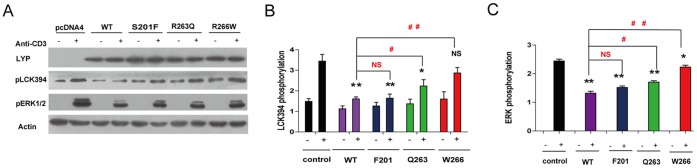
Effect of Lyp and 3 variants on T cell signaling. (A) Jurkat T cells were transfected with the His/Myc tagged full-length Lyp plasmids or mutated variants, and stimulated with medium or anti-CD3 (OKT3) antibody for 5 min. The level of full-length Lyp protein was detected by anti-Myc antibody. The phosphorylation of Lck394 and phosphorylation of ERK1/2 were monitored by immunoblotting with specific antibodies. Actin was detected as loading control. (B–C) Statistical analysis of Lck394 phosphorylation and ERK1/2 phosphorylation in Jurkat T cells overexpressed with Lyp wild type and variants. ** represents P<0.01 compared to the signals in control T cells stimulated with anti-CD3. ## represents P<0.01 compared to the stimulated signals in T cells overexpressed with wild-type Lyp.

To investigate whether the decrease of these variants’ phosphatase activity directly correlate to their functions, we transfected these mutants of full-length Lyp in human Jurkat T cells and looked at their effects on early T cell signaling. As shown in Fig. 4A, Lck and ERK were activated with stimulation of T cells with anti-CD3 antibody for 5 min in the control cells transfected with empty vector, while these activations were significantly blocked by overexpression of the Lyp wild type plasmid. Equal expression of Lyp mutants F201 and Q263 caused less but significant inhibition on Lck394 activation, while W266 failed to inhibit Lck394 activation compared with wild-type transfected cells (Fig. 4A & 4B). As Ras-MEK-ERK signaling axis was the major downstream following Lck activation, and was important for T cell proliferation, we next detected the ERK activation by monitoring ERK phosphorylation level at threonine 202 and tyrosine 204 positions. As shown in Fig. 4A and 4C, all Lyp variants and wild type blocked ERK activation, with a decreased ability in the order of: wild type> F201> Q263> W266. Among these, F201 variant was similar to wild type as a negative regulator in T cell signaling. Although Q263 only moderately decreased Lyp’s ability in inactivation of Lck, it displayed impaired ability in downregulation of ERK activation, which was in agreement with previous published study [25]. Compared to Q263, W266 was more defective in regulation of T cell signaling, which didn’t affect either Lck or ERK activation.

**Figure 5:**
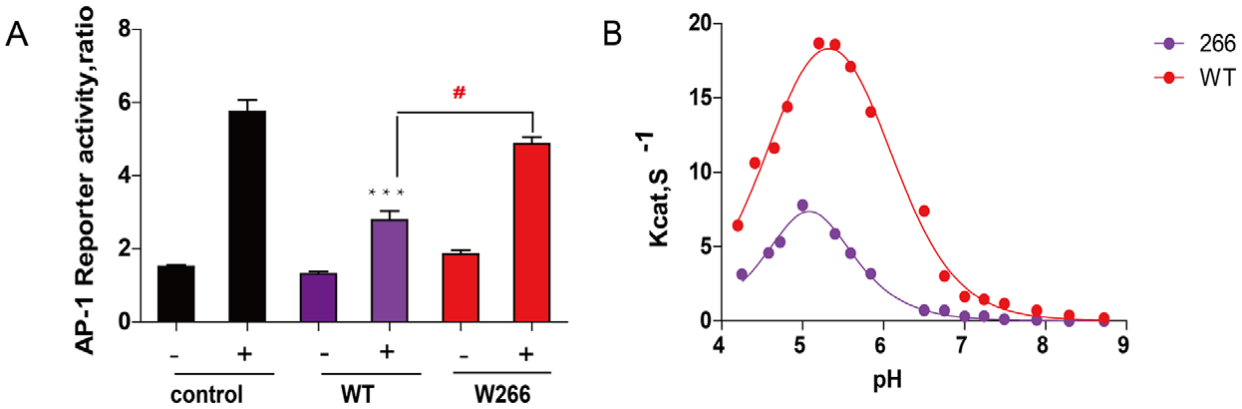
Lyp-W266 variant loses its function in T cell activation and its pH profile of enzyme activity. (A) Comparison of Lyp wild type and W266 variant on TCR-induced AP-1 transcriptional activity. (B) pH-Kcat profile of the pNPP hydrolysis catalyzed by Lyp wild type and R266W mutant.

Of the three Lyp variants tested, W266 greatly disturbed its activity both in T cell signaling and enzymatically. To provide further evidence that W266 is a loss-of-function mutant in T cell function, we measured the transcriptional activity of activator protein-1 (AP-1) in Jurkat T cells expressing full-length Lyp wild type or W266 variant. AP-1 transcription factor, which is a critical element involved in interleukin-2 production, is a downstream of ERK signaling [12], [30]. As expected, affter 6-hour anti-CD3 stimulation, a 4-fold increase in AP-1 luciferase activity was observed in Jurkat cells only transfecting the empty vector. In agreement of its function in early T cell signaling, overexpression of wild-type Lyp resulted in a 2.5-fold decrease in TCR-induced AP-1 activation, while W266 had impaired ability to attenuate AP-1 transcriptional activity (Fig. 5A). To investigate the underlying biochemical mechanism of functional loss of W266 variant, we measured pH dependence of W266 catalyzed pNPP hydrolysis compared with the wild type Lyp, as most PTPs use acid-base reaction mechanism for their catalysis [31], [32], [33], [34]. As shown in Fig. 5B, we observed bell-shaped Kcat-pH profiles for both Lyp wild type and W266 variant. The pK_ES1_ value of pH profile for W266-catalyzed pNPP reaction is 4.1±0.1, left shifted 0.17 on the acidic limb compared to that of the wild type Lyp, and the pK_ES2_ value of W266 is determined to be 6.1±0.1, left shifted 0.43 on the basic limb compared to that of the wild type. The observed pK_ES2_ mostly reflects the change of catalysis rate limiting step from E–P hydrolysis to E–P formation, in which the protonation of D195 is the key determinant of these steps [32]. Therefore, the results suggest that the W266 variant affects the protonated state of general acid D195 in Lyp, probably through changing its local environment.

## Discussion

Our studies revealed the Lyp variant S201F moderately decreased its phosphatase activity and ability in regulating T cell signaling, while R266W and R263Q significantly decreased their activities towards physiological substrates and behaved as loss-of-function mutants in T cells. As shown in Fig. 1C, S201 locates at the beginning of helix alpha 3 and is exposed to solvent. The substitution of S201 by F may induce it to fold inside of the structure due to its hydrophobic nature. Such a structural change could disturb the conformation of the WPD loop, thus affecting both the K2 and small substrates binding like pNPP (scheme 1). These observations could explain that variant 201F significantly decreased the Kcat toward pNPP. From the primary sequence alignment of 4 classic tyrosine phosphatases, Lyp, PTPN12, PTPN18 and PTP1B, S201 is a conserved residue, which agrees with the hypothesis that a hydrophilic residue was favored to maintain its intrinsic PTP activity (Fig. 1F). However, substitution of F at positon 201 only displayed limited effects on Kcat/Km of Lyp toward the phosphor-Lck 394 peptide and catalytic ability toward the phosphor-Src protein. Together, F201 is still an effective T cell signaling negative regulator. These observations suggest F201 maintained good activity towards its physiological substrate other than non-specific small substrate, probably a stricter substrate specificity compared with the wild type Lyp.

The R266 is a structually conserved residue among the protein tyrosine phosphatase superfamily. In the prototype tyrosine phosphatase PTP1B, a conserved R254 at equivalent position forms the bottom of the second “pY” binding pocket and has important structural roles in maintaining its phosphatase activity and substrate recognition. In agreement with our findings, the mutation of PTP1B R254Q has reduced Kcat toward pNPP by 17 folds and increased Km by 23 folds. The R254K mutant decreased its Kcat/Km value by 16 folds toward small substrate pNPP [35]. As shown in Lyp crystal structure, (Fig. 1E and 1F), the guanine group of the R266 forms two hydrogen bonds with carbonyls of main chain L272 and V273, to stabilize the conformation of Q loop, which harbors the highly conserved catalytic residue Q276 to correctly position the nucleophlic water for E-P hydrolysis [31], [36], [37], [38]. In addition, R266 also forms 2 hydrogen bonds with Ser 35 on Lyp specific insert, and hydrophobic interactions with L31 and V273 to tether the specific Lyp insert to the Lyp main fold. Substitution of R266 to W could retain some hydrophobic interactions for protein folding but would certainly lose the important hydrogen bonds which function to stabilize the Q loop conformation and tether the Lyp specific insert. As a result, we observed a significant decrease of Kcat on W266 towards pNPP and phosphor-peptide substrates, together with a functional loss in Jurkat T cell. A direct effect may come from change of Q276 position around active site and decreased K3 in the catalysis (scheme 1). In addition, once we measured the pH dependence catalysis of W266 polymorphism, the pKa of basic limb left shifted about 0.47, probably due to the change of the conserved general acid D195 protonation state.

Unlike F201 and W266, which draw few attentions in human diseases, the minor variant Q263 of Lyp has been identified to reduce the risk of several other autoimmune diseases except T1D, including SLE, RA and UC, but increase the susceptibility to infectious disease like PT. Our current results agree with previous finding that R263Q Lyp variant decreased its phosphatase activity and lost its ability in regulation of T cell signaling. Besides R263Q, we have found here that R266W variant of Lyp significantly impaired its phosphatase activity and capacity in regulation of T cell function. Whether R266W also reduces the risk of autoimmune diseases, such as SLE, UC and RA, and how it affects the susceptibility to PT are worth to investigate in future investigations. Such studies, together with inspections on association of more Lyp genetic variants with a broader spectrum of autoimmune diseases and infectious diseases, will help to evaluate Lyp as a drug target for autoimmune diseases.

## Materials and Methods

### Materials

Para-nitrophenyl phosphate (pNPP) was from Bio Basic Inc.(Canada). The 9 amino acid phosphor-peptide derived from the Y394 phosphorylation site of Lck was purchased from ChinaPeptides Co. (China). The Ni-NTA Resin was from Bio Basic Inc. The monoclonal anti-His, anti-Myc and anti-actin antibodies were from Santa Cruz Biotech. Anti-Src/pY416 antibody was purchased from Invitrogen. Polyclonal anti-ERK1/2, anti-phospho-ERK1/2 antibodies were from Cell Signaling. Anti-CD3 (OKT3) was from eBioscience. All other chemicals and reagents were from Sigma.

### Mutageneis

The Lyp mutants R263Q, R266W and S201F were generated by PCR reactions with the QuikChange site-directed mutagenesis kit from Stratagene. The PAGE- purified oligonucleotide primers were from Beijing Genomics Institute (China) and the sequences were as follows: S201F, CCATGATGTACCTTCATTCATAGACCCTATTCTTGAG; R263Q, GTTTTCAGTTTGATCCAGGAAATGCGGACACAG; and R266W, GATCCGGGAAATGTGGACACAGAGGC. All mutations were verified by DNA sequencing from Beijing Genomics Institute.

### Expression and Purification of Lyp Catalytic Domain and Mutant Proteins

The catalytic domain of Lyp (residues 1–294) with N- terminal His tag was prepared and used for in vitro study as before [14].

The His-tagged Lyp wild type and mutants were expressed in *E. coli* BL21 DE3. In general, 12 liters of Lyp transformed *E. coli* were cultured, induced by 0.3 mM IPTG at 25°C. After induction, the cultures were pelleted by centrifugation at 4000 rpm. The cell pellets were washed with buffer A (20 mM Tris, pH 8.0, 300 mM NaCl) and were resuspended in 120 ml of ice-cold buffer A. The bacterial pellets were sonicated on ice for 5 min, and the lysates were centrifuged at 10,000 rpm at 4°C for 1 hour. The supernatant was then collected and incubated with 4 ml of Ni^2+^-NTA resin for 2 hour at 4°C. The protein bound Ni^2+^-NTA beads were pelleted at 1,000 rpm for 6 min and the supernatant was discarded. The beads were washed three times for 5 min each at 4°C. The bound His-Lyp protein was finally eluted with a buffer containing 20 mM Tris pH 8.0, 300 mM NaCl and 200 mM Imidazole. The protein was further purified through CM Sefinose (BBI) with salt gradient elution. The low-salt solution contains 20 mM MES, pH 6.0, 100 mM NaCl, 1 mM EDTA and 2 mM DTT. The high-salt solution contains 20 mM MES, pH 6.0, 1 M NaCl, 1 mM EDTA and 2 mM DTT. After purified by CM Sefinose, the protein was further concentrated and stored at −80°C. Proteins were at least 99% pure by Coomassie staining after SDS-PAGE electrophoresis. Protein concentration was quantified by Bradford Protein Assay Kit.

The His-tagged Src catalytic domain construct was a gift from Dr.lefkowitz lab in Duke University. The Src protein purification and phosphorylation were performed as described previously [39].

### Enzyme Kinetics

Initial rate measurements for the Lyp-catalyzed pNPP hydrolysis was determined as described previously [12], [40]. All assays were performed at 37°C in 50 mM 3,3-dimethylglutarate (pH 7.0) buffer except for the experiment of pH dependences. 1 mM EDTA and 1 mM DTT were included in 50 mM 3,3-dimethylglutarate buffer, and the ionic strength of 0.15 M was adjusted with NaCl. Previous enzymological studies suggest that the PTPs catalysis have two steps [34], [41], shown in scheme1, in which ArOPO3 is the substrate. When pNPP was used as substrate, the reaction was stopped by addition of 1.0 M NaOH, and the activity was detected by monitoring the absorbance of paranitrophenol at 405 nm. The dephosphorylation of Lyp toward a 9 amino acid pTyr peptide Ac-EDNE(pY)TARE-NH2, or recombinant phosphorylated Src protein was carried out under the same conditions as pNPP. The reaction for phosphor-peptiede was stopped by addition of BIOMOL GREEN™ (ENZO) and the phosphate released was measured at 620 nm. The Lyp-catalyzed Src dephosphory- lation was stopped by 1 mM pervanadate and the SDS buffer, and the extent of reaction was analyzed by Western blot with anti-Src/pY416 antibody.

The pH dependence was carried out in the following buffers: 100 mM acetate (pH 4.3–5.0), 50 mM succinate (pH 5.0–6.0), 50 mM 3,3-dimethylglutarate buffer (pH 6.0–7.3), 100 mM Tris/HCl buffer (pH 7.5–8.7). All buffers contained 1 mM EDTA and 1 mM DTT, and was adjusted to an ionic strength of 0.15 M with NaCl. The Kcat value for pNPP hydrolysis catalyzed by the wild type Lyp and R266W mutant were determined at 37°C. To fit the Kcat value against pH, Equation 1 was used:
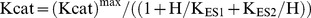
(1)


In this equation, K_ES1_ and K_ES2_ are the apparent ionization constants of enzyme-substrate complex in the rate-limiting step, and H is the proton concentration [31].

### Cell Culture, Transfection, and Immunoblotting Assay

Jurkat T cells were purchased from ATCC and grown at 37°C in RPMI 1640 medium supplemented with 10% FBS. Full-length Lyp wild type, S201F, R263Q and R266W mutants were subcloned into the pcDNA4/mycHis plasmid, and T cells were transfected with plasmids by electroporation with Neon (Invitrogen). After transfection, the cells were stimulated with 5 µg/ml anti-CD3 antibody (OKT3) or medium for 5 min. Subsequently, cells were lysed in 50 mM Tris, pH 7.5, 150 mM NaCl, 10 mM NaF, 2 mM EDTA, 10% Glycerol, 1% NP-40, 0.25% Sodium deoxycholate, 1 mM NaVO4, 1 mM PMSF, 0.3 µM aprotinin, 130 µM bestiatin, 1 µM leupeptin and 1 µM pepstatin. Cell lysates were subjected to denaturing SDS/PAGE, and transferred to nitrocellulose membrane. The Western blots were blocked with BSA, and immunoblotted by appropriate primary antibodies followed by incubation with HRP-conjugated secondary antibodies.

#### AP-1/TK-Renilla luciferase assay

Jurkat T cells overexpressed Lyp and luciferase were used here. 2×10^5^ cells were transfected by electroporation with 800 ng of the AP-1-luciferase plasmid, 40 ng of the Renilla-TK plasmid and 1 µg full-length Lyp wild type, R266W mutant or pcDNA4 vector. Forty-eight hours after transfection, Jurkat cells were stimulated with 5 µg/ml OKT3 or untreated for 6 hours. According to Promega’s instruction (Promega, Cat.No.E1960), dual luciferase activity was measured, and AP-1 transcriptional activity was normalized by Renilla activity.

#### Data analysis

Data analysis was conducted with Image J and Graphpad software. All data were presented as mean ± standard error of the mean, and statistical comparisons were made with ANOVA tests.

## Supporting Information

Figure S1 **Sequence alignment of Lyp from different species.** Residues of position 201, 263, 266 are highlighted in red. The unconservative residues are shown in blue.
(TIF)

Figure S2 **Coomassie blue staining of SDS-polyacrylamide gel of purified wild-type Lyp catalytic domain and variants.**
(TIF)
