# Supplementary figures and images for: Biochemical and Functional Studies of Lymphoid-Specific Tyrosine Phosphatase (Lyp) Variants S201F and R266W
Source: PLoS One. 2012 Aug 27;7(8):e43631. doi: 10.1371/journal.pone.0043631 (PMC3428364; doi:10.1371/journal.pone.0043631)

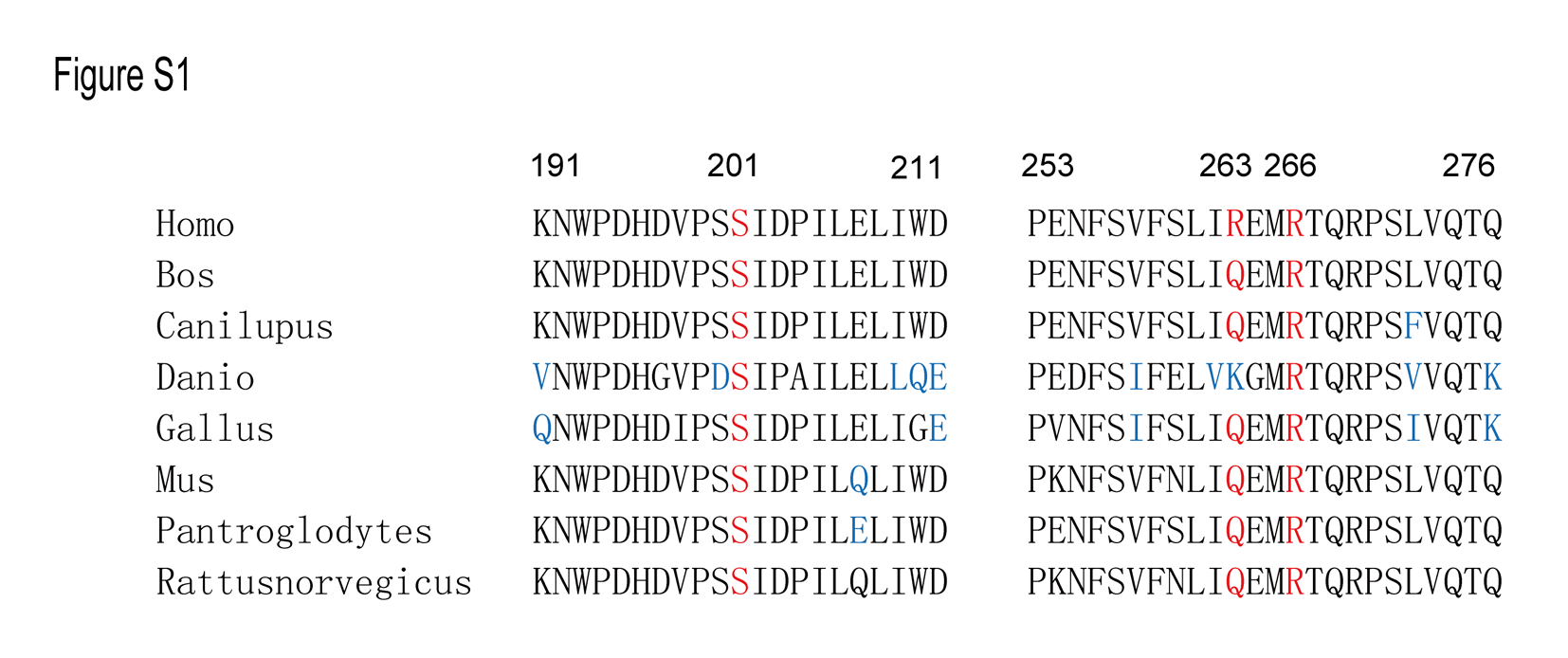

Supplement: Figure S1 — Sequence alignment of Lyp from different species. Residues of position 201, 263, 266 are highlighted in red. The unconservative residues are shown in blue. (TIF) [file pone.0043631.s001.tif]

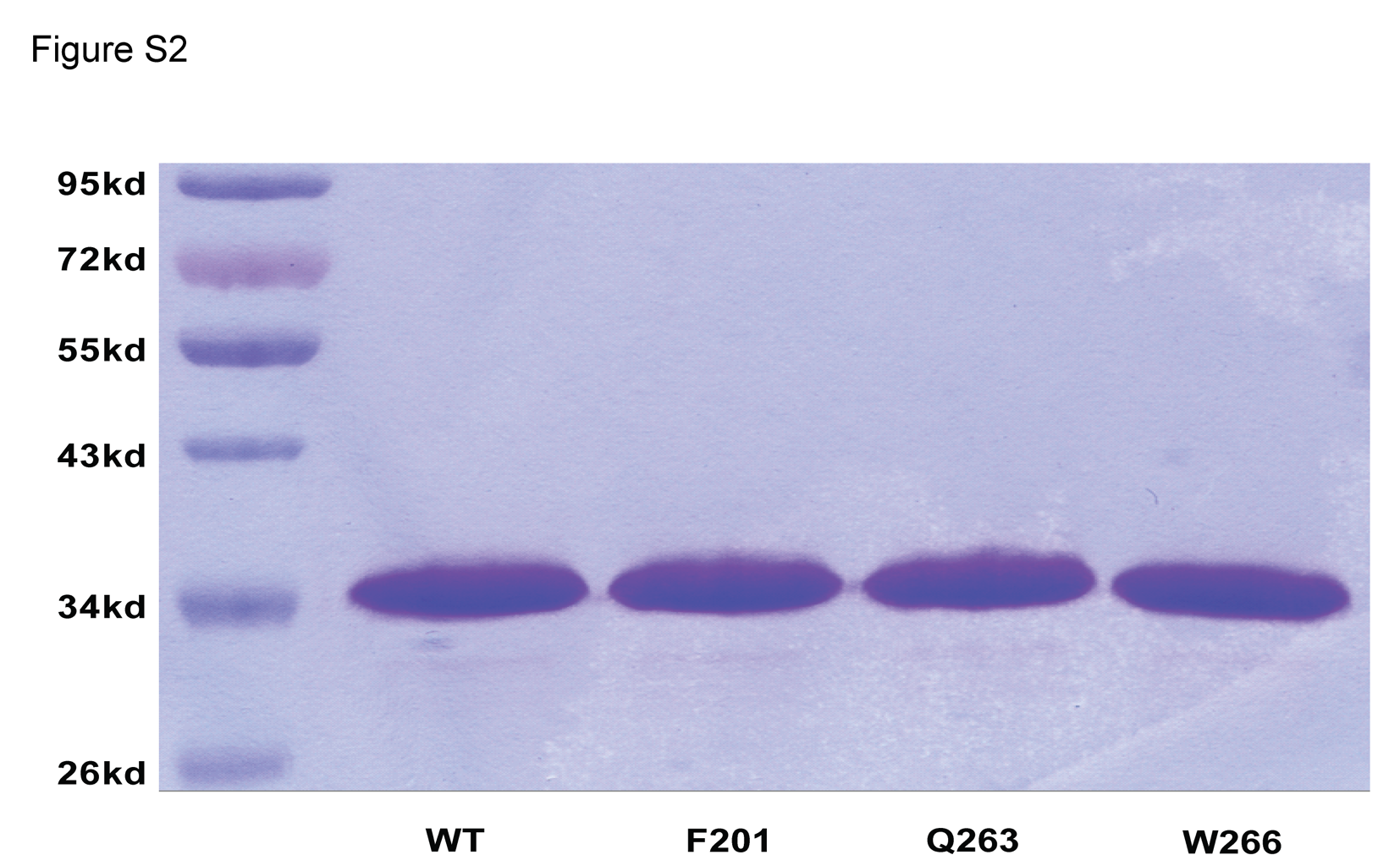

Supplement: Figure S2 — Coomassie blue staining of SDS-polyacrylamide gel of purified wild-type Lyp catalytic domain and variants. (TIF) [file pone.0043631.s002.tif]
